# Intravenous Immunoglobulin Therapy Administered Early after Narcolepsy Type 1 Onset in Three Patients Evaluated by Clinical and Polysomnographic Follow-Up

**DOI:** 10.1155/2018/1671072

**Published:** 2018-10-15

**Authors:** Elisabeth Ruppert, Hélène Zagala, Juliette Chambe, Henri Comtet, Ulker Kilic-Huck, François Lefebvre, Marc Bataillard, Carmen Schroder, Laurent Calvel, Patrice Bourgin

**Affiliations:** ^1^Department of Neurology, Sleep Disorders Center, CIRCSom, Hôpital Civil, 1 place de l'Hôpital, 67091 Strasbourg, France; ^2^Institute for Cellular and Integrative Neurosciences, CNRS-UPR 3212, University of Strasbourg, 5 rue Blaise Pascal, 67000 Strasbourg, France; ^3^Department of General Practice, School of Medicine, 4 rue Kirschleger, 67085 Strasbourg, France; ^4^Biostatistics Department, Hôpital Civil, 1 place de l'Hôpital, 67091 Strasbourg, France; ^5^Department of Child Psychiatry, Hôpital Civil, 1 place de l'Hôpital, 67091 Strasbourg, France; ^6^Department of Palliative Care, Hôpital Civil, 1 place de l'Hôpital, 67091 Strasbourg, France

## Abstract

Narcolepsy type 1 is a rare disabling sleep disorder mainly characterized by excessive daytime sleepiness and cataplexy, an emotion-triggered sudden loss of muscle tone. Patients have a selective degeneration of hypocretin-producing neurons in the dorsolateral posterior hypothalamus with growing evidence supporting the hypothesis of an autoimmune mechanism. Few case studies that reported intravenous immunoglobulin therapy (IVIg) suggest the efficacy of IVIg when administered early after disease onset, but the results are controversial. In these retrospective case observations, IVIg cycles were initiated within one to four months after cataplexy onset in a twenty-seven-year-old man, a ten-year-old girl, and a seven-year-old boy, all three with early onset typical narcolepsy type 1. Efficacy of treatment (three IVIg cycles of 1 g/kg administered at four-week intervals) was evaluated based on clinical, polysomnographic, and multiple sleep latency test (mean latency and SOREM) follow-up. Two patients reported decreased cataplexy frequency and ameliorated daytime sleepiness, but no significant amelioration of polysomnographic parameters was observed. Given the possibility of spontaneous improvement of cataplexy frequency with self-behavioral adjustments, these observations would need to be confirmed by larger controlled studies. Based on the present study and current literature, proof of concept is still missing thus prohibiting the consideration of IVIg as an efficient treatment option.

## 1. Introduction

Narcolepsy type 1 is a rare disabling sleep disorder mainly characterized by excessive daytime sleepiness and cataplexy, an emotion-triggered sudden loss of muscle tone [1, 2]. Patients have a selective degeneration of hypocretin-producing neurons in the dorsolateral posterior hypothalamus [3, 4] and about 90–95% of patients with typical narcolepsy-cataplexy have undetectable cerebrospinal fluid (CSF) hypocretin-1 levels [5]. Type 1 narcolepsy is strongly associated with the HLA DQB1^∗^06:02 allele coding for HLA class II molecules presenting antigenic peptides to CD4+ T-cells [6]. Growing evidence supports the hypothesis that the selective neurodegeneration results from an autoimmune mechanism [2, 7] providing a rationale for the use of immunomodulation therapy.

Only a few case studies have investigated the effect of intravenous immunoglobulin therapy (IVIg) with controversial data [8–19]. Some of these studies suggest a potentially disease-modifying effect of IVIg in reducing narcolepsy symptoms when administered early after disease onset. However, evaluations were mostly based upon subjective clinical elements. Few of these studies used polysomnography (PSG). We aimed to evaluate the efficacy of IVIg therapy in three patients with early onset typical narcolepsy type 1 using clinical and polysomnographic follow-up before and after each of the three cycles of IVIg.

## 2. Materials and Methods

These are three retrospective case observations. After obtaining patients' and parents' consents, three narcolepsy type 1 patients (2 children and 1 adult) were treated within 1 to 4 months after generalized cataplexy onset. Diagnosis of typical type 1 narcolepsy was clinically established in all patients, confirmed by polysomnography and multiple sleep latency tests (MSLT) <8 min with ≥2 SOREM, and undetectable CSF hypocretin-1 levels (<40 pg ml^−1^). All patients were positive for the HLA DQB1^∗^06:02 allele.

Patients were treated with IVIg at a dose of 1 g/kg, administered over 2 days in the adult and over 1 day in the children. Three IVIg cycles were realized at four-week intervals, according to the protocol used in previous investigations [10, 15–17]. Treatment efficacy was measured both clinically and objectively through PSG follow-up with overnight recordings and MSLT two weeks after each IVIg cycle. For each patient, all PSG evaluations were scored by the same technician blind to the therapeutic condition. Statistical analyses were carried out over the course of the different evaluations for each patient. Linear mixed-effects models were performed to take into account the repeated measurement of the outcomes. The significance level was set at 5%. All the analyses were made with R 3.0.2 software.

## 3. Results and Discussion

Patient number 1, a twenty-seven-year-old man, noticed the appearance of excessive daytime sleepiness (EDS) at the end of August 2013, 3 weeks after a flu-like syndrome. EDS increased gradually from one to three restorative naps per day. In the following month, he developed several partial cataplectic attacks per day mostly triggered by fear or anger. The first generalized cataplexy occurred beginning October. IVIg treatment was started by the end of October, within the month following cataplexy onset. The patient had gained 13 kg between EDS onset and PSG evaluation after the third IVIg cycle.

Patient number 2, a ten-year-old girl, presented EDS and irresistible naps that had appeared during July 2013 without any history of a triggering factor. EDS progressively increased during the summer, and generalized cataplectic attacks provoked by laughter appeared by the end of August with several episodes per day. IVIg cycles were initiated in October, within two months after cataplexy onset. The patient had gained progressively about 12 kg between EDS onset and PSG evaluation following the third IVIg cycle.

Patient number 3, a seven-year-old boy, presented with EDS and irresistible naps that had occurred at the end of May 2013, two weeks after a minor episode of rhinopharyngitis. He had also developed interrupted sleep, oppositional behavior, and irritability. Six weeks later, in July, a first cataplexy occurred, provoked by laughter. In September, at school, a restorative nap was implemented, improving both frequency and severity of symptoms. IVIg perfusions were initiated in November, within 4 months after generalized cataplexy onset. The patient had gained 6 kg over the first 6 months following EDS onset.

Patient number 3 was the only patient who had received an unadjuvanted H1N1 vaccine 4 years earlier that might be involved in triggering the disease.

IVIg infusions were overall well tolerated with some transient complaints of minor headache. The two children had infectious episodes, a flu-like syndrome for one and viral gastroenteritis for the other, leading to a few missed school days. Patient number 1 declared amelioration of EDS after the first IVIg cycle in a context where his professional activity changed from physically inactive to active and a preventive nap was progressively implemented. He also described being able to better handle cataplectic attacks, and generalized episodes lessened progressively from at least five per day at baseline to two or less than two per day after the first IVIg cycle and to two or three per week after the second IVIg cycle. Yet to the patient's opinion, there was no link between IVIg perfusions and EDS that persisted if he had no nap opportunity, as did cataplectic attacks. Partial attacks remained very frequent. For patient number 2, no clear amelioration of EDS nor cataplectic attacks were reported following the IVIg cycles. Reliable anamnesis was difficult in this child having a particularly complex social environment with substance abuse in parenthood. In patient number 3, the child's mother described clear amelioration of EDS, night sleep disruption, oppositional behavior, and cataplexy following the first IVIg cycle. On the other hand, amelioration had occurred progressively before baseline evaluation following a more adapted sleep hygiene. The child was described to control cataplexy behaviorally, avoiding situations that possibly could trigger cataplectic attacks and became more withdrawn (Table 1).

In none of the patients did PSG data show any significant changes regarding overnight sleep or MSLT using linear mixed-effects models (Supplemental Table 1, Table 1). We did not measure CSF hypocretin levels during follow-up.

All three patients were treated solely by IVIg from the initial visit date, and symptomatic treatment was only added after the evaluation following three months of IVIg treatment. Clinical follow-up at 6 months after baseline evaluation showed clear clinical efficiency regarding EDS and cataplexy with symptomatic treatment in patient number 1 (modafinil 400 mg/d, clomipramine 10 mg/d) and patient number 3 (modafinil 400 mg/d, sodium oxybate 5 g/d), whereas patient number 2 (modafinil 600 mg/d, clomipramine 50 mg/d) described some amelioration regarding EDS, but cataplexy remained uncontrolled. Due to the severe phenotype in patient number 2 and contraindication to sodium oxybate, modafinil was raised rapidly up to 600 mg/day (Table 1).

Clinical follow-up at one year in patient number 1 did not show any significant evolution as compared to the 6-month follow-up. There was no further follow-up as the patient had moved due to professional reasons. Patients number 2 and 3 are still followed up. Patient number 2 remains uncontrolled regarding EDS and cataplexy associated with an extremely poor sleep hygiene (no regular sleep-wake cycles, cosleeping, and noisy environment) and major overweight. No cataplectic episode was observed in patient number 3 following introduction of sodium oxybate, and EDS persists ameliorated. However, he complains of deteriorated sleep with two prolonged awakenings per night in a context of anxiety, night eating, and overweight associated with moderate obstructive sleep apnea.

Patients reported some amelioration of EDS and generalized cataplexy, yet objective PSG parameters showed no disease-modifying effect. Whether IVIg could help recover part of the hypocretin dysfunction involved in cataplexy pathophysiology remains speculative. Intriguingly, there is some discrepancy between cataplexy improvement and lack of amelioration regarding PSG parameters and EDS as measured by MSLT. Subjective evaluation of EDS by naps and its objective recording through MSLT is a further difficulty in evaluating symptom severity. These observations are difficult to interpret in the absence of controls matched for disease severity calling for larger samples studies. However, given the rarity of the disease, and especially the important latency between disease onset and patients being referred to sleep centers for diagnosis, larger controlled prospective studies are a challenge considering that only a few cases have been reported in the literature.

## 4. Conclusion

Based on the present study and current literature, proof of concept remains missing before considering IVIg as an efficient treatment option. This may result from an early severe hypocretin neuron loss at the time of symptom occurrence rendering IVIg treatment ineffective. Further research and development of therapeutic approaches to narcolepsy type 1 are needed to find a curative treatment for narcolepsy with possibly more emphasis on cellular rather than humoral immunosuppression therapies [20, 21].

## Figures and Tables

**Table 1:** Clinical and MSLT characteristics at baseline and under IVIg therapy.

Evaluation	Patient number 1—IVIg 1 month after cataplexy onset					Patient number 2—IVIg 2 months after cataplexy onset					Patient number 3—IVIg 4 months after cataplexy onset				
	Baseline	1 month	2 months	3 months	6 months of medication^∗^	Baseline	1 month	2 months	3 months	6 months of medication^∗∗^	Baseline	1 month	2 months	3 months	6 months of medication^∗∗∗^
Generalized cataplexy	>5/day	≤2/day	2/week	2/week	3/week	>5/day	≤3/day	>5/day	>5/day	>5/day	<1/day	<1/week	<1/week	<1/week	0
Naps	≤3/day	1/day	1/day	1/day	0	5-6/day	5-6/day	5-6/day	5-6/day	1-2/day	2/day	≤2/day	≤2/day	≤2/day	1/day
MSLT	5.8 min	1.6 min	2.1 min	2 min	NA	3.8 min	3.2 min	1.2 min	0.7 min	NA	8 min	2.9 min	2.6 min	9.2 min	NA
	3 SOREM	3 SOREM	3 SOREM	4 SOREM		4 SOREM	5 SOREM	5 SOREM	4 SOREM		2 SOREM	3 SOREM	5 SOREM	5 SOREM	

Clinical follow-up at 6 months was done under daily medication with ^∗^modafinil 400 mg and clomipramine 10 mg; ^∗∗^modafinil 600 mg and clomipramine 50 mg; and ^∗∗∗^modafinil 400 mg and sodium oxybate 5 g. NA: no available data.

## Data Availability

Readers may access the data underlying the findings of the study by contacting the corresponding author.

## Supplementary Materials

Supplementary Materials Supplemental Table 1: overnight polysomnographic characteristics at baseline and follow-up under IVIg therapy. H1 corresponds to baseline recordings, first (N1) and second (N2) nights; H2, H3, and H4 correspond to follow-up recordings two weeks after 1st, 2nd, and 3rd IVIg cycles. NA: no available data.
